# Performance evaluation of the Pima™ point-of-care CD4 analyser using capillary blood sampling in field tests in South Africa 

**DOI:** 10.1186/1758-2652-15-3

**Published:** 2012-01-30

**Authors:** Deborah K Glencross, Lindi M Coetzee, Mamsallah Faal, Martin Masango, Wendy S Stevens, WD Francois Venter, Regina Osih

**Affiliations:** 1 Department of Molecular Medicine and Haematology, Faculty of Health Sciences, University of the Witwatersrand, 7 York Road, Parktown, 2198, Johannesburg, South Africa; 2 Wits Reproductive Health and HIV Institute, University of the Witwatersrand, Esselen Street, Hillbrow, 2198, Johannesburg, South Africa; 3 National Health Laboratory Service of South Africa, 7 York Road, Parktown, 2198; 4 Society for Family Health "New Start" HCT (HIV counselling and testing) programme, South Africa

## Abstract

**Background:**

Point-of-care CD4 testing can provide immediate CD4 reporting at HIV-testing sites. This study evaluated performance of capillary blood sampling using the point-of-care Pima™ CD4 device in representative primary health care clinics doing HIV testing.

**Methods:**

Prior to testing, prescribed capillary-sampling and instrument training was undertaken by suppliers across all sites. Matching venous EDTA samples were drawn throughout for comparison to laboratory predicate methodology (PLG/CD4). In Phase I, Pima™ cartridges were pipette-filled with EDTA venous blood in the laboratory (N = 100). In Phase II (N = 77), Pima™ CD4 with capillary sampling was performed by a single operator in a hospital-based antenatal clinic. During subsequent field testing, Pima™ CD4 with capillary sampling was performed in primary health care clinics on HIV-positive patients by multiple attending nursing personnel in a rural clinic (Phase-IIIA, N = 96) and an inner-city clinic (Phase-IIIB, N = 139).

**Results:**

Pima™ CD4 compared favourably to predicate/CD4 when cartridges were pipette-filled with venous blood (bias -17.3 ± STDev = 36.7 cells/mm^3^; precision-to-predicate %CV < 6%). Decreased precision of Pima™ CD4 to predicate/CD4 (varying from 17.6 to 28.8%SIM CV; mean bias = 37.9 ± STDev = 179.5 cells/mm^3^) was noted during field testing in the hospital antenatal clinic. In the rural clinic field-studies, unacceptable precision-to-predicate and positive bias was noted (mean 28.4%SIM CV; mean bias = +105.7 ± STDev = 225.4 cells/mm^3^). With additional proactive manufacturer support, reliable performance was noted in the subsequent inner-city clinic field study where acceptable precision-to-predicate (11%SIM CV) and less bias of Pima™ to predicate was shown (BA bias ~11 ± STDev = 69 cells/mm^3^).

**Conclusions:**

Variable precision of Pima™ to predicate CD4 across study sites was attributable to variable capillary sampling. Poor precision was noted in the outlying primary health care clinic where the system is most likely to be used. Stringent attention to capillary blood collection technique is therefore imperative if technologies like Pima™ are used with capillary sampling at the POC. Pima™ CD4 analysis with venous blood was shown to be reproducible, but testing at the point of care exposes operators to biohazard risk related to uncapping vacutainer samples and pipetting of blood, and is best placed in smaller laboratories using established principles of Good Clinical Laboratory Practice. The development of capillary sampling quality control methods that assure reliable CD4 counts at the point of care are awaited.

## Background

CD4 lymphocyte counts are used in HIV-positive patients for initiation of antiretroviral therapy (ART), to direct empiric treatments of suspected opportunistic infection [1,2] and to identify patients failing therapy in resource-poor settings [3]. In South Africa during 2010, the national Department of Health embarked on a widespread voluntary HIV counselling and testing (HCT) initiative to drastically extend its national HIV/AIDS ART programme across the country: almost 12 million people were tested (18% of these HIV positive) in 12 months [4]. The South African National Health Laboratory Service (SA-NHLS) [5] currently supports an extensive network of CD4 laboratories [6] to service this increased need for testing across the country.

Easy to use, accessible and simplified technologies for CD4 cell count testing at the point of care (POC) have long been anticipated. Provision of an ideal, fully accessible and decentralized near-patient CD4 count method [7,8], which is also rapid, reliable, robust and affordable, has nevertheless remained a challenge. Despite reservations of pathology testing at the POC [9,10], the prevailing notion remains that provision of CD4 counts in the context of voluntary counselling of patients for HIV/AIDS could improve enrolment of eligible HIV-positive patients onto ART programmes [11-15].

The Pima™ CD4 Analyser (Alere, South Africa), described elsewhere [11,16], is a light, portable POC CD4 instrument proposed for such near-patient CD4 testing. The aim of this study is to report on the outcomes of the performance of the Pima™ CD4 analyser in adults using either venous blood samples in the laboratory or capillary blood sampling in typical South African primary health HCT clinics where such technology may be used, and compare performance of the Pima™ against laboratory-based, state-of-the-art flow cytometric predicate CD4 methodology [6,17].

## Methods

### Study description: phases of testing

This prospective validation of the Pima™ POC CD4 analyser (Alere: http://www.alere.com) was coordinated through the SA-NHLS Charlotte Maxeke Johannesburg Academic Hospital (CMJAH) CD4 reference laboratory, located in Johannesburg, South Africa, and performed according to the "Standards for Reporting of Diagnostics Accuracy" (STARD) [18]. Ethics approval for this study was obtained through the University of the Witwatersrand (protocol #M10116). Pima™ CD4 testing was performed in adult patients only.

*Phase I *was designed to assess baseline inherent accuracy and precision of the instrument in a controlled laboratory environment. During this phase of testing, Pima™ cartridges were pipette-filled with a fixed volume (20 mm^3^) of well-mixed venous K_3_EDTA anti-coagulated blood taken from consecutive samples sent for routine predicate CD4 testing (< 24 hours old, N = 100) at the SA-NHLS CMJAH laboratory.

In *Phase II *(N = 91), the impact of Pima™ testing using manufacturer-prescribed capillary blood sampling was evaluated in the same hospital (CMJAH) at the antenatal HCT clinic using two of the Pima™ instruments validated during Phase I testing (Pima #2 and #3). To eliminate possible variation introduced with varying sample age, Phase II was specifically designed to facilitate laboratory testing on fresh samples (matched venous EDTA samples for both predicate and Pima™ were tested within one to two hours of venesection). Pima™ testing in the clinic was performed by a single nursing sister, thereby avoiding variability potentially introduced by multiple operators. A direct comparison of Pima™ to Pima™ performance using matched venous and capillary blood samples was also performed (N = 77).

In *Phase III*, the Pima™ system was "field" tested. This phase included two parts. In Phase IIIA (N = 96), testing was undertaken independently through a local donor-funded group in two Limpopo province primary health care HCT clinics (semi-rural, less resourced), where finger-prick sampling was routinely performed for other pathology testing (typically HIV rapid testing). Here, the Pima™ testing was performed by multiple operators attending their respective patients; this is an important perspective to assess performance in typical HCT testing environments. Matching K_3_EDTA samples were drawn simultaneously by phlebotomy staff and the samples referred for comparative predicate CD4 testing at the nearest SA-NHLS CD4 laboratory.

In *Phase IIIB *(N = 139), the Pima™ system was tested in an inner-city primary health care HCT clinic in Johannesburg. Testing was performed by two nursing attendants. Matching K_3_EDTA samples were also drawn simultaneously by phlebotomy staff and the samples sent for comparative predicate CD4 methodology testing at the nearby SA-NHLS Reference CMJAH CD4 laboratory. In this last phase of evaluation, following less favourable outcomes noted in Phase II and IIIA field testing, additional manufacturer-driven training and support was given to operators to assess whether a proactive approach to manufacturer support could improve field performance seen in Phase IIIA.

The same lancet (Sarstedt Safety Lancet, 1.5 mm Blade/1.6 mm depth, designated as "lancet 1", n = 87) was used for capillary sampling across all clinical phases of testing. In the last sub-study (Phase IIIB), it was tested alongside a different lancet (Carelet Blue Safety Lancet/1.5 mm Blade/2.00 mm depth, designated "lancet 2", n = 52) to assess whether lancet type had contributed to the poorer Pima™ performance noted in the previous clinical phases.

### Testing of samples

Across the clinic sites and in the SA-NHLS CMJAH laboratory respectively, Pima™ operator training for either nursing personnel (Phases II and III) or dedicated study scientists (Phase I) was undertaken by the suppliers prior to commencing testing, according to methods defined by the manufacturer. Reporting of either Pima™ or predicate CD4 results was blinded in the clinics and the NHLS laboratories respectively. All patients who had signed consent to participate in the study were recruited on a first-come, first-serve basis for Phases II and III of this study. Error codes and other instrument error logs were noted by operators to establish the overall error rate of each instrument used (Tables 1, 2, 3).

**Table 1:** Laboratory Pima™ testing using venous blood

Phase I EDTA venous blood	Pima #1	Pima #2	Pima #3	Pima #4	All Pima DATA
N	53	61	59	58	100**

Range of CD4 counts*	2-1297	4-1217	5-1190	7-972	4.5-1235

Mean CD4 count*	349	334	354	317	318

**%Similarity to Predicate** *(**%SIM **Mean ± SD)*	99.26 ± 5.34	99.15 ± 5.35	99.64 ± 6.42	97.17 ± 5.01	98.17 ± 4.57

**%SIM CV**^*#*^	5.38%	5.40%	6.44%	5.16%	4.66%

BA Bias* ± 1 STDev (PIMA - PLG) **(95% CI of bias mean)**	-14.38 ± 37.9 (-23.8 to -3.9)	-11.05 ± 42.4 (-21.9 to 0.19)	-13.6 ± 53.3 (-27.5 to 0.22)	-22.6 ± 40.3 (-33.2 to -12.0)	-17.3 ± 36.7 (-24.0 to -10.03)

BA 95% LOA*	-88.7 to 59.9	-94.1 to 72.1	-118.2 to 90.8	-101.6 to 56.4	-89.4 to 54.7

Statistical analyses across four Pima™ analysers tested in the laboratory during Phase 1 using a fixed 20 mm^3 ^volume of manually pipetted EDTA venous blood and compared against predicate flow cytometry (PLG/CD4). All values, with the exception of N, refer to CD4 cell counts in cells/mm^3^. BA = Bland-Altman statistical analysis. LOA = limits of agreement. #Precision of Pima™ to Predicate method expressed as %SIM CV.

*cells/mm^3^

**The average Pima/CD4 value was used for comparison to predicate CD4 where more than two simultaneous Pima values were obtained.

**Table 2:** Hospital antenatal Pima™ field testing using capillary blood

Phase II	Laboratory Pima™ vs. Predicate			Clinic Pima™ vs. Predicate		
	**Venous EDTA blood**			**Capillary blood**		

	*Pima #1	*Pima #4	*Mean **Pima #1 and #4**	**Pima #3**	**Pima #2**	**All values**

**N**	91	91	91	43	34	77

Range of CD4 counts*	32 - 1186	23 - 1299	28 - 1243	28 - 1092	50 - 1056	28 - 1092

Mean CD4 count*(median)	385.6 (341.0)	399.8 (357.0)	392.7 (347.0)	335.0 (405.0)	350.2 (290.0)	380.8 (329.0)

**%Similarity to Predicate** *(**%SIM **Mean ± SD)*	96.9% ± 7.89	98.3% ± 7.05	97.6% ± 6.5	98.6% ± 28.45	96.1% ± 16.8	98.7 ± 23.02

**%SIM CV**^*#*^	8.1%	7.2%	6.7%	28.8%	17.6%	23.3

BA bias* ± 1 STDev (PIMA - PLG) **(95% CI of bias mean)**	-26.6 ± 73.3 (-41.9 to -11.4)	-12.4 ± 68.1 (-26.6 to 1.8)	-19.6 ± 66.1 (-33.3 to -5.8)	-31.79 ± 213.1 (-97.4 to 33.8)	-45.5 ± 127.7 (-90.1 to -1.0)	-37.9 ± 179.5 (-78.3 to 2.87)

BA 95% LOA*	-170.4 to 117.1	-145.8 to 121.0	-149.1 to 110.0	-147.4 to 385.9	-295.8 to 204.6	-389.1 to 309.8

Statistical analysis of Pima™ analysers field-tested in a hospital-based antenatal HCT clinic (N = 77) and in the laboratory (N = 91) during Phase II testing versus predicate reporting. A direct comparison of Pima performance using matched venous vs. capillary blood samples is shown. All values, with the exception of N, refer to CD4 cell counts in cells/mm^3^. BA = Bland-Altman statistical analysis. LOA = limits of agreement. #Precision of Pima™ to Predicate method expressed as %SIM CV.

*cells/mm^3^

**Table 3:** Primary health care clinic field testing using capillary blood

PHASE III	Rural clinic Pima™ (3 sites) versus Predicate (Phase IIIA)		Inner-city clinic Pima™ (1 site) versus Predicate (Phase IIIB)			
	
	PREDICATE	Pima™	PREDICATE	Pima™ *Lancet 1* *(Sarsedt)*	PREDICATE	Pima™ *Lancet 2* *(Caralet Blue)*
**N**	96	96	87	87	52	52

Range of CD4 counts*	2 - 1871	31 - 2747	2 - 1604	1 - 1358	3 - 966	2 - 968

Mean CD4 count*(median)	617.5 (492.0)	719.4 (677.5)	350 (302.0)	359.0 (305.0)	268.4 (255.5)	257.2 (238.0)

**% Similarity to Predicate** *(**%SIM **Mean ± SD)*	114.8% ± 32.6		101.4% ± 10.1		98.6% ± 11.2	

**%SIM CV**^*#*^	28.4%CV		10.0%		11.3%	

BA bias* ± 1 STDev (PIMA - PLG) **(95% CI of bias mean)**	+105.7 ± 225.4 (60.1 to 151.4)		+8.9 ± 112.3 (-14.9 to 32.8)		-11.2 ± 69.3 (-30.5 to 8.1)	

BA 95% LOA*	-336.0 to 547.0		-211.1 to 229.0		-147.0 to 124.6	

Statistical analysis of for Pima™ analysers field-tested in Phases IIIA and IIIB across two rural (N = 96) and one inner-city HCT clinic (N = 139), respectively, versus predicate reporting. All values, with the exception of N, refer to CD4 cell counts in cells/mm^3^. BA = Bland Altman statistical analysis. LOA = limits of agreement. #Precision of Pima™ to Predicate method expressed as %SIM CV.

*cells/mm^3^

Panleucogated (PLG) CD4 testing, the predicate methodology of the SA-NHLS, described elsewhere [6,17], was used as the reference standard throughout this study. All PLG CD4 testing was performed by SA-NHLS technologists working in the routine diagnostic service, according to Good Clinical Laboratory Practice (GCLP) and the Standard Operating Procedures of the SA-NHLS [5].

### Quality control

Daily Pima™ analyser quality control (QC) was performed with manufacturer-supplied, reusable, bead-filled cartridges with pre-defined counts (high and low controls) before commencing daily testing. This QC was performed for the duration of the study prior to sample testing, both in the laboratory (during Phases I and II) and on all analysers used in the field (Phases II and III).

Accuracy and precision of predicate PLG/CD4 testing in both SA-NHLS laboratories was established by daily monitoring of instrument stability (FlowCheck™, Beckman Coulter, Miami, Fl), through further method and system performance verification (Immunotrol™, Beckman Coulter, Miami, Fl) [19,20], as well as ongoing continuous intra-sample quality bead-rate monitoring [6,21]. The CMJAH SA-NHLS CD4 laboratory participates in the CD4/AFREQAS [22] and the UK NEQAS [23], while the district SA-NHLS laboratory participates in the CD4/AFREQAS. Both laboratories are South African National Accreditation Service [24] accredited.

### Process verification of Pima™ testing

**Figure 1:**
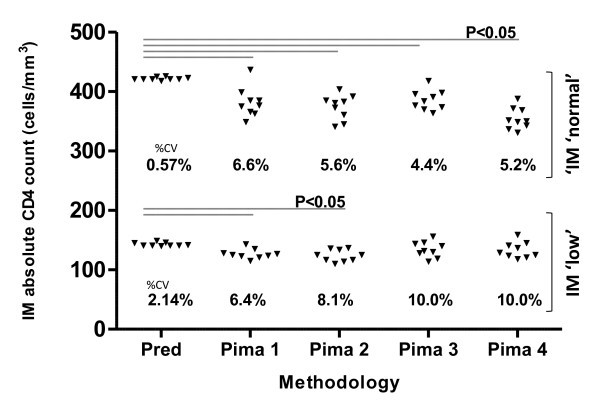
**Daily quality control**. Figure to show excellent reproducibility using stabilized whole blood quality control material, i.e., IM "normal" and IM "low" across both predicate (PLG/CD4) and four Pima™ analysers during laboratory testing in Phase I. Statistically significant differences (Student's t-test, p < 0.05) between predicate and Pima™ CD4 counts, shown in the figure, reflect tight precision-to-predicate demonstrated in the %SIM analysis. No statistical differences were, however, noted between individual Pima™ analysers when testing either IM normal or IM low material (one-way ANOVA, p > 0.05). Performance using external quality control material is shown in Figure 2.

Also, during Phase I, additional process verification controls, "Normal" and "Low" Immunotrol™ (BC, Miami, FL), were tested daily for nine consecutive days on the Pima™ instruments in the laboratory (Figure 1). For this analysis, 20 mm^3 ^of stabilized whole blood material (Immunotrol™, Beckman Coulter, Miami, FL) was manually pipetted into separate Pima™ cartridges (Figure 1) and analyzed individually on each instrument.

#### External quality assessment of Pima™ and predicate CD4 testing

**Figure 2:**
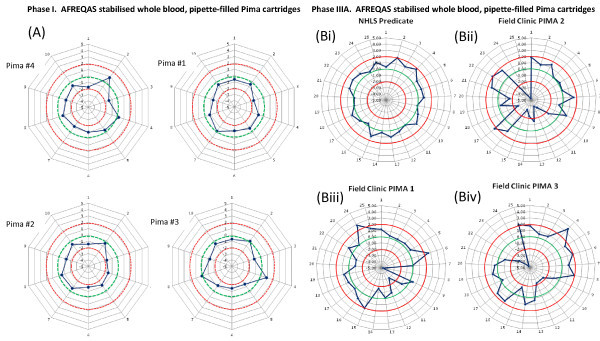
**External quality assessment using retrospective AFREQAS samples**. Figure to show performance of Pima™ analysers using panels of retrospective SA-NHLS AFREQAS stabilized blood samples to establish external quality assessment of testing. In (A) performance of four Pima™ analysers - Pima™ analysers #2 and #3 were subsequently used for field testing in Phase II and in (B) during Phase III, the SA-NHLS Predicate (SA-NHLS Polokwane using PLG/CD4 - performance is noted together with performance of three independent Pima™ analysers field tested using pipette-filled cartridges on site. See details in text. Red circles define acceptable limits of ± 2 SDI and the green circle represents ideal performance (SDI = 0). Actual performance of the respective laboratory/clinic is shown in blue.

Specifically assembled panels of archived SA-NHLS CD4 AFREQAS [22] stabilised blood samples were used to further verify accuracy and precision of SA-NHLS predicate testing, as well as being tested across four Pima™ instruments tested in the laboratory during Phase I (Figure 2A). In the Phase IIIA field study (Figure 2B), similar extended panels were used for external quality assessment of the three field Pima™ instruments, as well as the SA-NHLS laboratory performing comparative predicate testing.

The panel results obtained by Pima™ or predicate method were compared with the pooled global mean CD4 results obtained through participation of all users of the respective trials [25] to establish retrospective external quality assessment. A standard deviation index (SDI) or z-score value was calculated for each trial sample tested, with acceptable limits expected at between -2 and +2 SDI per result. Performance was represented in modified radar plots as previously described [22,25] for ease of comparison between instruments in Phase I or sites during Phase IIIa.

### Statistical analysis

All statistical analyses and graphic display of data were generated with GraphPad Prism™ Software Version 5.03. Basic statistics, including minimum, maximum, median and mean values, standard deviation (STDev) and percentage coefficient of variation (%CV) for respective data sets were calculated where applicable. Absolute CD4 results from both predicate (PLG/CD4) and by Pima™ were compared using the % Similarity (%SIM) model [26] with the derived %CV of the %SIM used as a measurement of precision-to-predicate. All %SIM values were corrected to 100% (similar) if by predicate testing, the CD4 result was < 100 cells/mm^3^. In the %SIM model, method agreement is adequate where %SIM calculated values fall between 95% and 105% and where precision of test-method to predicate-method (%SIM CV) is shown to be < 8%. Bland-Altman analyses [27], with the bias (difference) defined as [Test minus Predicate], were used to indicate bias and agreement between technologies. Where indicated, to assess true differences at lower CD4 counts without the influence of higher CD4 counts in the data sets, data was grouped into two categories: CD4 count of < 350 cells/mm^3 ^and therefore eligible for treatment by World Health Organization guidelines [28]) and CD4 count of > 350 cells/mm^3^, the level of patients who are generally ineligible for treatment in the SA programme (except in the presence of AIDS-defining opportunistic illnesses).

The non-parametric t-test and Mann-Whitney test were used to establish possible significance of differences between predicate and Pima™ CD4 results. Comparison of two or more groups of data was performed using one-way ANOVA for non-parametric tests, with Kruskall Wallis testing used to establish a p value and Dunn's Multiple Comparisons test used to indicate significant differences between groups, where applicable.

## Results

### Pima™ daily quality control

During Phase I, documented bead-cartridge quality control analysis over time (26-32 days) reflected excellent instrument precision (%CVs of < 2.5%) irrespective of instrument used (Pima #1-4). Review of the bead QC revealed cartridge-bead "low"/"high" counts of 152/995, 150/961, 149/989 and 221/828 beads/mm^3 ^for Pima instruments #1-4, respectively, with longitudinal low/high QC reproducibility (CV%) of 1.4%/1.6%, 1.9%/0.6%, 2.1%/0.7% and 1.9%/1.1% noted. Control cartridge within-instrument precision testing, performed at least once for each instrument in a replicate set of 10 bead analyses during Phase 1, yielded similar results to the longitudinal reproducibility we have described, with low/high bead QC %CVs noted at 2% or less. Similar QC bead cartridge precision was shown throughout other phases of testing (data not shown). Of note, specifically in the rural field clinic HCT during Phase IIIA, Pima™ instrument bead precision was excellent and noted at 2.88%CV and 1.81%CV for "low" and "high" bead control cartridges, respectively (suggesting acceptable instrument performance in the context of the poorer sampling testing performance subsequently noted at this site).

Process-verification QC of both the predicate methodology, as well as individual Pima™ analysis (Figure 1) was also performed during Phase I using the commercially available stabilised blood product, Immunotrol™ (IM, Beckman Coulter, Miami, FL). IM "normal" and IM "low" materials (manufacturer range 422 ± 165 and 142 ± 18 cells/mm^3 ^respectively) were analysed for nine consecutive days on both the MC-XL flow and across four Pima™ instruments (Figure 1). For IM "normal" and IM "low" material, this analysis revealed excellent day-to-day precision of predicate CD4 testing (PLG-CD4 MC-XL) with %CV 0.5% and CV 2.0% noted, respectively. Acceptable precision was seen across four Pima™ analysers, with average %CV of 6.2% and 9.1% noted, respectively (Figure 1).

Bland-Altman bias analysis confirmed the negative bias noted in the %SIM analysis, i.e., -47.8 ± STDev = 23.5 cells/mm^3 ^(IM "normal") and -13.3 ± STDev = 11.7 cells/mm^3 ^(IM "low"). In other words, the predicate CD4 counts read higher than Pima™, but were still within the defined limits of the IM product package insert. Further testing using a panel of 10 retrospective AFREQAS (stabilised) blood samples performed in Phase I revealed similar overall under-reading by Pima™, with all Pima™ results falling within acceptable 2SDI limits (Figure 2A). Despite underestimation of CD4 counts by Pima™ instruments when either stabilised blood product was tested in comparison with predicate results (%SIM decreased between 92% and 95%), differences noted were relatively constant, as reflected by the tight precision of similarity of Pima™ CD4 to predicate methodology (%SIM CV < 5%). These differences were not regarded as clinically significant.

### Phase I: evaluation of Pima™ performance using K_3_EDTA venous blood

Results of Phase I testing with venous blood (N = 100) is shown in Table 1. Overall, a consistent and very tight negative bias with small variation around the bias was noted (-17 ± STDev = 36.7 cells/mm^3 ^and Bland Altman (BA) 95% limits of agreement of -89.4 to 54.7 cells/mm^3^). Excellent agreement between methods was noted in the %SIM analysis with an average 98% ± STDev 4.6% (range 97.7% to 99.6%) and very tight precision-to-predicate demonstrated (mean %SIM CV < 7%). During this phase, between-instrument precision simultaneously tested across two (N = 8), three (N = 25) or four Pima™ analysers (N = 24) was excellent and showed no statistically significance difference in CD4 counts between Pima™ instruments (p > 0.05, one-way ANOVA): between-analyser precision was calculated at ~9.4%CV across four instruments. There were no "no-read" errors noted in the laboratory evaluation.

### Phase II: impact of capillary blood sampling on performance of Pima™ at the POC in a hospital based antenatal clinic

**Figure 3:**
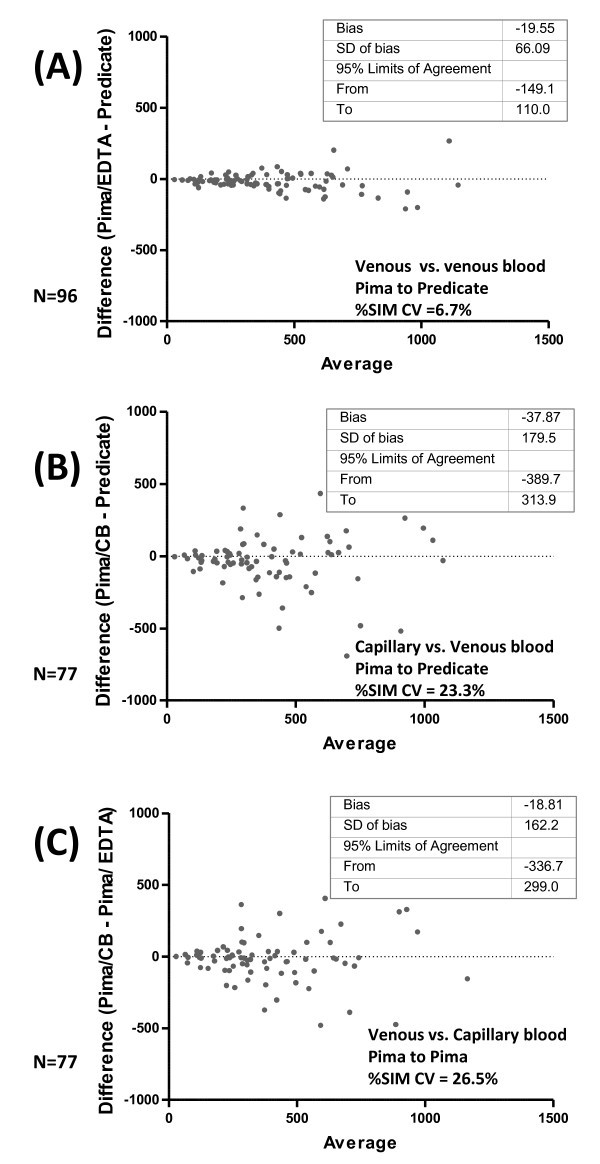
**Pima™ performance with venous and capillary blood sampling**. In (A), Predicate and Pima™ are both tested using venous EDTA blood, revealing excellent method agreement and small, clinically insignificant bias between systems. In (B), decreasing precision of Pima™ precision-to-predicate CD4 is noted where capillary blood sampling is used. The mean bias is within clinically acceptable limits; however, the variation noted around the mean bias is poor, with unacceptably wide LOA noted (range > 600 cells/mm^3^). In (C), further decreasing precision-to-predicate is revealed with even higher %SIM %CV noted in a direct Pima™ to Pima™ comparison of capillary versus venous blood. Although overall BA bias between sampling methods is clinically insignificant, the variation of this bias (reflected in the 95% LOA) is unacceptably wide, confirming the poor %SIM CV of 26.5%. Bland-Altman values shown above are cells/mm^3^. CB = capillary blood.

A larger negative bias, with wider variation around this bias, was documented between Pima™ CD4 and predicate CD4 counts (Table 2, Figure 3B) in an antenatal primary health care (PHC) clinic where a single operator performed the testing using capillary sampling. The %SIM analysis (Figure 3B) confirmed loss of precision in relation to that seen when venous blood was tested by Pima (Figure 3A), with corresponding %SIM CV decreasing to17.6% and 28.8% for each of the two instruments used in the clinic, respectively (average calculated 23.3CV%; see Table 2 and Figure 3B for details). "No read" errors were noted in five of 48 (10.4%) and nine of 43 (20.93%) samples tested on each of the respective instruments used.

Also during Phase II, the impact of capillary blood sampling on Pima performance was highlighted in a direct comparison of Pima™ to Pima™ using matched capillary and venous blood samples taken from the same patient and tested at the same time. Poor precision (%SIM CV = 26.5%) between the two sampling methods was revealed (Figure 3C). As expected [29,30], the BA analysis revealed a moderate mean bias of -18 cells/mm^3 ^between sampling methods. However, wide BA limits of agreement (LOA) revealed significant sample to sample differences, varying from -336.7 to 299.0 cells/mm^3^.

### Phase III: "field" testing

During Phase III, the Pima™ was "field" tested in a semi-rural PHC clinic (termed Phase IIIA, where multiple operators participated), followed by assessment in a better-resourced inner-city clinic (termed "Phase IIIB", where only two operators performed testing). At the rural site, of the 111 samples drawn, eight could not be tested in the laboratory as the samples were clotted on receipt and were excluded from the final analysis. A further seven of the 111 (6.8%) samples did not have matching Pima™ results due to "no read"/"invalid" CD4 readings recorded at the clinic sites (ethics clearance did not allow for repeat capillary sampling to be performed). In the final comparison of 96 matching CD4 results (see Table 3 for details), Bland-Altman bias analysis revealed an overall substantial, clinically significant difference to predicate reporting (105.7 to ± 225.4 cells/mm^3^) with very wide limits of agreement revealed (95% LOA -336.0 to +547.4 cells/mm^3^).

**Figure 4:**
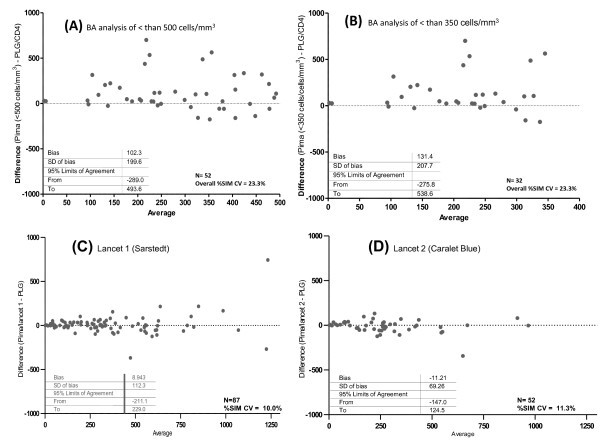
**Field testing of Pima™**. Figure to show Bland-Altman analysis of Pima™ performance during field testing. In Phase IIIA, comparison of Pima™ CD4 versus predicate is shown in a rural PHC HCT clinic (N = 96) where (A) includes samples of < 500 cells/mm^3 ^and (B) samples of < 350 cells/mm^3^. In Phase IIIB, comparison of Pima™ CD4 versus predicate is shown in an inner-city PHC HCT clinic (N = 139), revealing acceptable performance irrespective of whether (C), lancet 1 (N = 87) or (D) lancet 2 (N = 52) was used. Bland-Altman values shown above are in cells/mm^3^.

A sub-analysis done within the 96 sample set to investigate this bias in the CD4 count range less than 500 cells/mm^3 ^(Figure 4A) or less than 350 cells/mm^3 ^(Figure 4B) similarly revealed very wide mean BA bias of 102.3 ± STDev = 199.6 and 131.4 ± STDev = 207.7 cells/mm^3^, respectively. Although counts were lower, a wider LOA in the < 350 cells/mm^3 ^group revealed increasing error in this clinically relevant CD4 range (Figure 4A and 4B). Further analysis of the outlying results in this group to assess possible misclassification of patients' eligibility for ART, revealed that 10 out of 32 patients would have missed an opportunity for ART initiation if tested by Pima™ on site, with 16 of 32 recording a difference of Pima™ CD4 to predicate CD4 of > 30% (50.0% of patients tested). Further, in those patients with CD4 counts of greater than 350 but less than 500 cells/mm^3^, 23.5% (four of 17) had significantly higher CD4 counts by Pima™ testing than by predicate methodology and would ultimately have missed the opportunity for therapy intervention. A further eight of 17 (41.7%) patients were shown to have POC Pima™ CD4 results with > 30% difference to predicate testing. Percentage similarity analysis of the full data confirmed these findings, with Pima™ reading higher than predicate (114.8 ± 32.6%) and %SIM CV reflecting the wide variation of Pima reporting relative to predicate reporting (average 28.4%CV).

The bead cartridge quality control performed on the Pima™ instruments used at these sites however reflected acceptable baseline instrument reproducibility (n = 10, % CV of 2.8% and %CV of 1.8% for low and normal bead-cartridge analyses, respectively), confirming operator influence. Testing of the AFREQAS panel performed after the initial field study, where AFREQAS QC material was manually pipetted into cartridges by operators, served to confirm inherent reliability of the Pima™ instruments used at these clinics; a few outlying results noted on this analysis across the three instruments used in the field clinics (Figure 2Bii, iii and 2iv) reflecting inexperience with pipetting technique. Importantly, testing of the same 25-sample AFREQAS panel by the regional SA-NHLS laboratory demonstrated acceptable and reliable external quality assessment performance of the NHLS laboratory that performed the comparative predicate technology (Figure 2Bi).

During the last phase of testing (Phase IIIB), the performance of the Pima™ instrument was evaluated in an inner-city PHC HCT clinic (Table 3, Figure 4C and 4D). "No read" errors were reported in 14 of a total of 153 samples tested (final N = 139). In striking contrast to the Phase IIIA findings, the %SIM analysis showed considerably less bias and tighter LOA variance, irrespective of whether lancet 1 (N = 87, 101.4% ± STDev = 10.1%) or lancet 2 (N = 52, 98.6% ± STDev = 11.2%) was used. Adequate precision-to-predicate method was also demonstrated (%SIM CV noted at 10.0% and 11.3% for lancets "1" and "2", respectively).

## Discussion

The study was undertaken to assess the performance of the Pima™ POC CD4 instrument in a primary health care HCT clinic environment using manufacturer-prescribed capillary blood sampling. The study involved testing on adult patients only and did not include children younger than 18. In this regard, the Pima™ analyser only reports absolute CD4 counts and cannot be used where CD4% of lymphocyte values are required used for pediatric clinical assessment.

Overall, Pima™ performance varied markedly between field study sites and was shown to depend on the quality of the capillary blood sampling (overall field study %SIM CVs varied from 11.3 to 28.3%, see Tables 2, 3 and Figures 3, 4.). The Bland-Altman analysis (LOA) revealed wide variation to predicate counts, varying from acceptable limits (BA LOA, -147.0 to 124.6 cells/mm^3 ^in the inner-city study site) to unacceptable limits in a rural primary health care environment where multiple attending nursing staff, trained and practiced in finger-prick sampling for rapid HIV testing performed both capillary blood sampling and Pima™ analysis (BA LOA, -336.0 to 547.0 cells/mm^3^). The %SIM CV confirmed this varying precision of Pima™ to predicate counting, varying from acceptable levels (10.0%CV to 11.3%CV) to unacceptable levels (varying from 17.6%CV to 28.8%CV) in field clinics. This finding was consistent across sub-studies performed (phases II and III), irrespective of whether a single user (Phase II) or multiple users (Phase III) had performed the testing (detail in Tables 2 and 3). Similar variation has noted in peer-reviewed literature (also see additional file 1).

Similar variation and poor precision was also noted when matched Pima™ to Pima™ evaluation of venous versus capillary samples were tested, further confirming the negative impact of (poor) capillary blood sampling on Pima™ performance (Figure 3C). These results are contrasted by excellent Pima™ performance noted in a controlled laboratory setting [31] when venous blood was used for Pima™ testing (%SIM CV 4.7%, see Tables 1, 2 and Figure 3A).

The Pima™ field data from the rural primary health care setting is of particular concern as it reflects a scenario where the Pima™ analyser is most likely to be used in the context of HIV and PHC clinics. Several aspects warrant discussion in this context. First, problems related to capillary sampling are likely to be universal and not unique to Pima™ analysis: any small device that uses capillary sampling is likely to be affected by such sampling error. Proactive and continuous supplier training did result in an improvement of performance (Phase IIIB/inner-city clinic) but still less than that noted with venous blood. Intensive and ongoing training has been recommended [16,32,33] to improve performance in this context. Such an approach may not, however, be sustainable in the longer term across a multitude of sites, especially if instruments are used in PHC facilities situated in less accessible, rural areas. Substantial human resources will be needed going forward to provide continuous training and supervision to ensure proper capillary sampling at the POC, especially where multiple operators may be required to use the system, as noted in our rural field study, further complicated by problems of high staff turnover [34].

Several factors may have played a role in the variable testing precision noted here and in other related studies [16,33,35]. Some non-operator dependent biological factors may have possibly affected capillary sampling include viz. possible rapid clotting of capillary blood [36,37]: Pima™ reporting was acceptable in at least one site, suggesting that clotting of samples is unlikely to have caused poor precision. Sampling type per se is excluded: capillary blood parameter values are not expected to vary from values in venous blood [38], certainly not in relation to haematological counts [29] and after discarding the first drop of blood [30].

Differences to predicate counts (variability) noted here are therefore most likely attributable to differences in quality of sampling. In this context, the terminology ("finger-prick") itself is confusing. Capillary blood sampling is different from typical "finger-prick" bleeding. Lack of attention to testing protocols and inconsistent or absent quality control has been previously reported during HCT at the POC with the much simpler "rapid HIV test", which uses true finger-prick sampling [39]. The sampling required for Pima™ CD4 testing is different and more akin to a classic haematological "bleeding time". It is therefore possible that lack of insight into the difference between finger-prick and capillary bleeding methods contributed to variability noted here, especially in those clinics where operators claim familiarity with "finger-prick" techniques used for HIV rapid testing. Reinforcement about differences between finger-prick and capillary sampling is therefore vital: the importance of absolute strict adherence to prescribed manufacturer methods of sampling to ensure adequate precision and accuracy of the Pima™ CD4 reporting at the POC cannot therefore be overemphasised.

Some inadequate cartridge filling, related to poor sampling, may have also contributed to the poorer performance noted in the rural clinics. Higher percentages (up to 23%) of "no read" readings have been similarly noted elsewhere [40]. Lancet type as a reason for poorer performance was excluded: no difference in performance was noted when the recommended Sarstedt/lancet 1 or the alternative Caralet Blue/lancet 2 was used.

The second point pertains to use of quality control material and the separate need for a quality control method to ensure quality of the capillary bleed. Although we (Figures 1 and 2), and others [35], have shown that process control or external quality assessment material can be used in the Pima™ analyser, such use only monitors inherent instrument performance and does not provide quality control of the most variable aspect of near-patient testing, the capillary bleed, which is crucial to ensure individual sample accuracy. Currently, this elusive and variable aspect of POC testing is the biggest challenge to ensuring quality CD4 counts at the POC. Research to develop a system for monitoring quality control of capillary sampling by operators is therefore desperately needed, especially in a primary health care environment where operator handling can negatively impact upon Pima™ results (or any other instrument at the POC that uses capillary sampling).

Third, the best use of the technology in its current format, bearing in mind the current limitations of poor capillary sampling quality revealed in our field studies, pertains to the idea that POC CD4 technologies, used in conjunction with HCT, will identify patients eligible for treatment [7,13,41]. We have shown variable precision, which causes either under- or in the case of our rural PHC field study, significant over-reading by Pima™ (BA bias was noted at +105.7 cells/mm^3 ^and confirmed by the %SIM model of 114.8 ± 32.6%). In our study, patients who would have therefore missed the opportunity for ART at a 350 CD4 cell/mm^3 ^cut-off (where Pima™ read falsely higher than predicate CD4) accounted for about 31% of participants in the rural PHC study. Here, more than 50% of patients tested showed a more than 30% difference (negative or positive bias) to predicate count. Other published studies have reported less numbers of patients misclassified (lost) for ART: 6.7-14% [16], 5.2% [35] or 17% [33].

In this context, local studies reveal very small and slow rises of CD4 counts after commencement of ART (only reaching median levels around 350 cells/mm^3 ^levels at three years post ART, [42]). Monitoring of such small patient responses to ART may therefore be both difficult and unreliable if such CD4 technologies with limited precision were also used to follow up patients' response to ART. This translates to mean that Pima™ POC CD4 testing or any POC system with reduced precision-to-predicate is best used for screening only, and in conjunction with better quality controlled, more robust, laboratory-based CD4 testing for patient follow up. This will ensure that overall, long-term, follow-up patient care is not compromised, especially if CD4 counts are used to predict treatment failure [3,43].

The alternative in the PHC HCT context where CD4 testing is to be used at the POC is to consider venous draw-based Pima™ testing. Manipulation of venous blood samples does, however, unfortunately expose the operator to biohazard risks associated with working with blood samples and may not be a practical option in all HCT clinics. Operators would require, at minimum, a dedicated area for sample testing and some training in GCLP [44] and need to be trained to uncap and recap vacutainer tubes and pipette venous blood into Pima™ cartridges. Such a within-clinic testing facility is possible [41,45], but requires dedicated staff to run the service: such testing is probably more appropriately placed in a smaller or mini-laboratory where such safety precautions can be implemented as routine practice. Despite the obvious disadvantage of testing venous blood at the POC, patients may prefer a venous blood draw.

CD4 counting is not the only pathology testing [33,46] required for staging and assessing eligibility of patients for ART. At minimum, a haemoglobin value, along with AST and ALT testing, cholesterol and urea and electrolyte testing, are required to assess eligibility before ART can be commenced. Here, repeated finger-pricks required to test each parameter individually on separate machines are likely to be uncomfortable and not readily accepted by patients: the idea of a single venous draw to perform multiple POC pathology testing (for testing in a PHC mini-lab or referral to a local nearby laboratory) may still be a more practical and acceptable option to patients, especially if they are required to return to the clinics anyway for adherence counselling before commencing ART [41].

The fourth point relates to costs of providing CD4 counts at the POC. Systems like Pima™ are likely to cost significantly more than conventional flow cytometry testing. Reagents/lancets for Pima™ testing are estimated at about US$10 per test, excluding other so-called hidden costs including quality control bead consumables, instrument maintenance and associated nursing personnel labour costs. Pima™ analysers and the required printers are quoted separately at more than $6000. In contrast, certainly in South Africa, predicate laboratory-based testing is charged in an all-encompassing fee for service of about $7-8 per test: this is fully inclusive of instrument placement and maintenance, reagents, labour and laboratory overheads. In such a scenario, POC CD4 testing could potentially therefore more than double current costs of conventional lab-based testing, depending on how widely it is implemented.

Recent studies show only modest increases in the proportion of patients who had received CD4 results at the POC who are subsequently successfully enrolled onto ART (from 12% to 22% [47] or 33% versus 47% [41]: loss to follow up remains high despite these early interventions. Bearing this in mind, and considering that resources may be better spent on improving existing laboratory infrastructure to improve all pathology services across a national health service, a tiered approach to service implementation [8] is envisaged through the SA-NHLS National Priority Programme. This would range from high-volume, centralised and consolidated laboratory testing [6] through local district laboratory servicing to fully decentralised POC testing in areas with very limited or no laboratory servicing [8], integrated with all other pathology services. This model, based on mapping existing primary health and HCT clinics to current existing laboratory infrastructure, potentially extends services to many more national laboratories to provide CD4 and other relevant and appropriate pathology servicing required for patient staging for ART. This approach maximises analyser service delivery and most effectively uses current funds and resources to ensure overall equitable access to CD4 and other pathology testing, irrespective of geographic location or clinic resource.

## Conclusions

In this study we have shown that, during field testing, the quality of capillary blood sampling negatively impacted on the precision and accuracy of the Pima™ point-of-care CD4 analyser. Capillary sampling therefore demands absolute diligence and stringency of sampling technique. Ongoing dedicated training as well as implementation of systems for monitoring and evaluation of testing is strongly recommended. Additional, properly controlled cost-benefit field studies [46], independent field studies and financial modelling studies to ensure best and efficient use of resources are, however, still needed. A true POC CD4 test for initiation of ART in an ideal dipstick version, which is both accurate and reliable, as well as cheap, is still eagerly awaited [48,49].

## Competing interests

DKG, LMC and WSS, employees within the National Health Laboratory Service (SA-NHLS), declare that the international patents rights for the method (PLG/CD4) used as a predicate and reference standard in this study are wholly owned by their employer, the South African NHLS. The remaining authors declare that they have no conflicts of interest.

## Authors' contributions

DKG undertook project conception and design, was involved in the analysis and interpretation of data and drafted and critically revised the manuscript. LMC performed analysis and interpretation of data and Pima™ laboratory testing. MF and MS were local study coordinators and were responsible for organizing patients and collating data. WDFV, WSS and RO gave input into the conception and design of the project and critically revised the manuscript. RO additionally organized ethics clearance for this study. All authors gave their final approval of the version to be published.

## Supplementary Material

Additional file 1 **Review of relevant peer-reviewed literature of related Pima™ studies and other comparative technologies**. The table contains data taken from recent peer-reviewed publications where Pima™ is compared to various predicate or alternative CD4 methodologies. Here, although in many instances mean bias between compared technologies is relatively small and acceptable, it is important to point out that these bias values are frequently not quoted in the context of actual method agreement. Here, attention is specifically drawn to the ranges of the 'limits of agreement' of the BA mean bias, which reflect the actual variation of differences noted between the technologies compared and give an idea of the precision of the methodology or new analyser tested in relation to that of the reference predicate method. For ease of comparison between studies, results have therefore been categorised into three groups according to the range of the Bland Altman limits of agreement. i.e., where the range is narrow and acceptable, and falls below 200 cells/mm^3 ^(highlighted in blue), those studies that reveal greater than 200 cells/mm^3 ^but less than 300 cells/mm^3 ^limits (highlighted in grey), and those studies that show very wide limits of agreement, greater than 300 cells/mm^3 ^(highlighted in red) and where corresponding %SIM %CV is shown to exceed 10%). Of note especially, is that the laboratory-based cluster within the group that show narrow BA limits of agreement, i.e., limits of agreement less than 200 cells/mm^3^. Click here for file
